# Multimodal Dimension Reduction and Subtype Classification of Head and Neck Squamous Cell Tumors

**DOI:** 10.3389/fonc.2022.892207

**Published:** 2022-07-13

**Authors:** Jonathan E. Bard, Norma J. Nowak, Michael J. Buck, Satrajit Sinha

**Affiliations:** ^1^ Department of Biochemistry, Jacobs School of Medicine and Biomedical Sciences, State University of New York at Buffalo, Buffalo, NY, United States; ^2^ Genomics and Bioinformatics Core, Jacobs School of Medicine and Biomedical Sciences, State University of New York at Buffalo, Buffalo, NY, United States; ^3^ Department of Biomedical Informatics, Jacobs School of Medicine and Biomedical Sciences, State University of New York at Buffalo, Buffalo, NY, United States

**Keywords:** multimodal, integration, multiomics, squamous cell carcinoma, classification

## Abstract

Traditional analysis of genomic data from bulk sequencing experiments seek to group and compare sample cohorts into biologically meaningful groups. To accomplish this task, large scale databases of patient-derived samples, like that of TCGA, have been established, giving the ability to interrogate multiple data modalities per tumor. We have developed a computational strategy employing multimodal integration paired with spectral clustering and modern dimension reduction techniques such as PHATE to provide a more robust method for cancer sub-type classification. Using this integrated approach, we have examined 514 Head and Neck Squamous Carcinoma (HNSC) tumor samples from TCGA across gene-expression, DNA-methylation, and microbiome data modalities. We show that these approaches, primarily developed for single-cell sequencing can be efficiently applied to bulk tumor sequencing data. Our multimodal analysis captures the dynamic heterogeneity, identifies new and refines subtypes of HNSC, and orders tumor samples along well-defined cellular trajectories. Collectively, these results showcase the inherent molecular complexity of tumors and offer insights into carcinogenesis and importance of targeted therapy. Computational techniques as highlighted in our study provide an organic and powerful approach to identify granular patterns in large and noisy datasets that may otherwise be overlooked.

## Introduction

Significant efforts have been made over the years to better characterize and partition tumors into biologically and molecularly meaningful distinct subtypes. The rationale behind such tumor classification was to enable more precise, effective, and targeted therapeutic strategies. Such efforts have been rewarded, as in the case for breast cancer where death rates have dropped by 39% since 1989 (1), in part due to accurate subtype classification leading to efficacious treatments (2). This is particularly the case for the highly heterogenous group of triple negative breast cancers, where better understanding of the complex tumor microenvironment has facilitated tailored treatment regimens, including effective immunomodulation therapies (3, 4).

Similar subtype classification has been attempted for other tumor types with varied success. For example, head and neck cancers (HNSC) are clinically defined using the tumor-node-metastases (TNM) classification system and more recently the AJCC/UICC staging system, which rely heavily on pathological features such as primary tumor characteristics, lymph node spread, and distant metastasis, as well as relevant clinical history like smoking and alcohol usage (5, 6). Recent inclusion of using p16^INK4A^ status for HPV+ Oropharyngeal cancer in the eighth edition of TNM guidelines (7, 8) and PDL-1 expression paired with tumor mutational burden prior to treatment with pembrolizumab (9) represents examples where biomarkers have proven to be valuable for better diagnosis and therapy. Despite these advances, for many of the patients with advanced HNSC, histological and clinical staging do not correlate with clinical responses or prognosis (10). Therefore, continued efforts in subtype classification with the goal of more precise targeted therapeutics based on molecular signatures are needed.

Data mining of the TCGA datasets, primarily those based on transcriptomic signatures has resulted in subtyping of carcinomas from various anatomical sites. In the case of HNSC, tumors have been classified into four primary groups, Atypical (HPV+), Classical, Mesenchymal, and Basal subtypes (11, 12). However, given that HNSCs are inherently diverse and complex diseases of profound inter- and intra-tumoral heterogeneity, it is likely that additional subtypes exist. Support for such molecular diversity of HNSC comes from the discovery of a distinct subtype with impaired H3K36 function based on DNA methylation states (13) and from a meta-analysis approach that integrated multiple datasets (14). In general, classification of HNSC (and similarly other tumors) has by and large relied on a single facet of tumor biology, such as global gene expression profile, methylation patterns, somatic mutation states, or HPV infection status (15). Although such single-data driven approach has improved identification and our overall understanding of tumor subtypes, it is likely that application of an integrated bioinformatics-driven method that takes into account multiple data modalities might offer a powerful tool for new discoveries (16, 17).

Here, we establish a generalizable approach to integrate multimodal datasets for tumor subtype classification. Using HNSC as an example, we have performed a *de novo* integrated analysis of the bulk gene expression, methylation array, and microbiome datasets from TCGA Squamous Carcinoma patients. Specifically, we utilized multimodal spectral clustering followed by uniform manifold approximation and projection (UMAP), and potential of heat diffusion for affinity-based transition embedding (PHATE) dimension reduction (18–20). Our results demonstrate that data integration, followed by two-dimensional projection of the integrated similarity matrix perform robustly in partitioning samples, while preserving biological significance. Importantly, we show that during this process, the full range of molecular heterogeneity of HNSC tumors are captured, avoiding information loss. Furthermore, PHATE dimension reduction captures a dynamic gradient of expression in HNSC, ordering samples along a cellular trajectory toward more invasive Squamous Cell Carcinoma (SCC) as evident by tell-tale gene expression profiles. These results have broad implications for the challenge of accurate cancer subtype prediction by providing a robust strategy of multimodal data integration, leading to more accurate subtyping, with the potential for guiding future therapeutic intervention.

## Materials and Methods

### Pan-Squamous Transcriptomic Expression Data Acquisition and Analysis

RNA expression profiles for each cohort were accessed using the gdc.cancer.gov portal for Hoadley et al. (21). RNA-expression data for HNSC, LUSC, ESCA, BLCA, and CESC were extracted from the batch corrected matrix file labelled EBPlusPlusAdjustPANCAN_illuminaHiSeq_RNASeq.v2.geneExp.tsv, resulting in a 1,925-sample x 20,531-gene matrix. This expression matrix was then passed into the UMAP and PHATE algorithms to derive 2-dimensional projections, using default parameters. To derive cross-cohort clusters, the expression matrix was supplied into the R package Spectrum v1.1, producing 9 clusters. For UMAP dimension reduction, the uwot v0.1.11 package was used with default parameters on the expression matrix. For PHATE dimension reduction the phateR v1.0.7 algorithm was used with default parameters on the pan-SCC rna-expression matrix.

### HNSC Transcriptomic Expression Data Acquisition and Analysis

For the HNSC specific analysis, a 514 samples x 20,531 gene matrix and associated metadata was constructed in R from the RSEM batch corrected file labelled “data_RNA_Seq_v2_expression_median.txt” available for download in CBioPortal (22). Pairwise statistical analysis utilized the R package rstatix v0.7.0 was used to preform Wilcoxon rank sum tests for changes in expression between clusters, correcting for multiple testing using the Holm-Bonferroni method. Data scaling for visualization was preformed using the base R function scale. Kaplan-Meier curves were generated using the R packages Survival v3.2-11 and Survminer v0.4.9. Overall survival status for each patient were provided as input and time-to-median survival outcomes were calculated. The sum of the normalized expression for mesenchymal associated genes (*FN1*, *VIM*, *ZEB1*, *CRS*, *TWIST2*, *SNAI2*, *CDH2*) and epithelial markers (*CLDN4*, *CLDN7*, *TJP3*, *PEMT*, and *CDH1*) was subtracted from the expression sum of basal markers (*SLC2A*, *SLC16A1*, *H1F1A*, *LAMC*, *COL17A1*, *ITGB1*, *AREG*, *EGFR*, *CDH3*, *KRT16*, *KRT17*, and *KRT14*) to compute a basal composite score, a similar strategy to that of Salt et al. (23).

### HNSC Illumina 450k Array Methylation Data Acquisition and Filtering

Per-sample methylation data was retrieved from the GDC data portal using the gdc-client command line utility. This dataset is comprised of 580 HNSC samples (528 solid tumor, 2 tumor metastatic, and 50 adjacent normal tissue), level 3 TCGA data release. Samples with associated transcriptomic data were subset and 514 solid tumors were chosen for follow-up analysis. Probes associating with or targeting SNPs, as well as sex-chromosome associated probes were filtered from the analysis per Papillion-Cavanagh et al. *NSD1*/H3K36 mutation group labels were taken as provided in **Supplemental Table 5** from Papillion-Cavanagh et al. (13). Our filtration cascade resulted in a 514-sample x 310,325-probe matrix used for subsequent analysis.

### HNSC Microbiome Data Acquisition

Per-sample microbiome measurements produced by Poore et al. were downloaded from CBioPortal using the file labelled “data_microbiome.txt” (24). This data is the result of the batched corrected Voom-SNM Kraken workflow described by Poore et al, with all putative contaminants removed (24). Samples with associated transcriptomic and methylation profiles were subset, resulting in a 514-sample x 1406-genus log2 CPM matrix which was used for subsequent integration analysis.

### HNSC Multimodal Integration Using Diffusion of Tensor Product Graphs

Methylation, transcriptomic, and microbiome data matrices were ordered by patient-sample ID, representing 514 head and neck solid tumors with complete multi-omic profiles. Each data modality was next introduced as an independent matrix to a self-adapting density-aware kernel developed by John et al. to produce a single sample-to-sample similarity matrix (25). This strategy dynamically accounts for the local density of k-nearest-neighboring samples, effectively amplifying the similarity between tumors with highly-similar profiles. Next, the three-resulting single-view derived similarity matrices are linearly combined using kernel addition prior to diffusion of the tensor-product graph (25, 26).The resulting similarity matrix (514x514) is then treated as input into subsequent spectral clustering by Spectrum, and for UMAP and PHATE dimension reduction analysis.

### HNSC Dimension Reduction Analysis and Data Visualization

UMAP dimension reduction analysis was performed using the R package uwot v0.1.11, using default parameters (20). PHATE dimension reduction analysis was preformed using the R package phateR v.1.0.7 (19). Default parameters for UMAP were used. For PHATE dimension reduction, the t = 200 was chosen, which controls the power of diffusion. For all dimension reduction techniques, the resulting 2-dimensional embeddings were extracted and stored in an R data frame associating sample names, spectral cluster assignment, clinical features, and X,Y coordinate. Visualizations were generated using the R package ggplot2 v3.3.5, ggpubr v0.4.0, and cowplot v.1.1.1 packages, and clinical variables, expression profiles, methylation beta values, and microbiome quantifications were overlayed. Clustering was performed using the R package Spectrum, using the multimodality gap method (method=2) and kernel tuning (tunekernel=t) parameters (25).

### Code Availability and Workflow

A graphical depiction of our analysis workflow for the panSCC and focused HNSC analysis is shown in **Supplemental Figure 16**. Analysis code is available at https://github.com/jebard/multimodal-tcga-hnsc. Clustering results and sample assignments can be accessed in CBioPortal as virtual study cohorts of the larger TCGA PanCancer HNSC 2019 set utilizing the following link: https://www.cbioportal.org/comparison/overlap?comparisonId=60804602e4b0242bd5d4984c.

## Results

### Dimension Reduction of TCGA PanCancer Transcriptomes Provide Efficient and Dynamic Representation of Tumor Samples

Large datasets such as bulk RNA-seq of tumors in TCGA have been traditionally analyzed by hierarchical clustering, k-means clustering, and matrix factorization approaches like principal component analysis. These techniques, though powerful, have some inherent limitations due to the high-dimensional, often-sparse and noisy nature of such datasets, and thus may miss non-linear relationships hidden in the complex data (27, 28). Such limitations prompted the development of non-linear computational methods such as t-SNE and UMAP (20, 29) to overcome the challenges associated with the massive amount of data and the excess noise level when it came to analysis of single-cell RNA sequencing (scRNA-seq) outputs. These methods have succeeded in providing intuitive and biologically meaningful displays by representing the high-dimensional scRNA-seq data in a low-dimensional space, while preserving the relevant local structure of the data. Indeed, there has been a growing interest in application of dimension reduction and visualization strategies to efficiently and accurately model many large-scale data types (30). We intuited that these robust techniques such as those developed for single-cell analysis could potentially be re-purposed and applied to large-scale bulk tumor datasets such as those generated by the TCGA project. We initially examined RNA-seq data from 1,925 tumors from 5 different anatomical sites, including lung (LUSC), head and neck (HNSC), esophageal (ESCA), cervical (CESC), and bladder cancers (BLCA) to test and evaluate the scalability of dimension reduction strategies on bulk sequencing datasets. These samples were chosen since they represent a broad range of tumors of primarily epithelial origin that share common gene-expression patterns and constitute a molecularly distinct pan-SCC cohort (31, 32).

For our analysis, we first decided to test two dimension-reduction techniques, Uniform Manifold and Approximation and Projection (UMAP) and Potential of Heat-diffusion for Affinity-based Transition Embedding (PHATE). While UMAP was chosen due to its wide-spread adoption across single-cell analysis pipelines, PHATE was selected since it offers visualization that preserves the local and global structure of the data, denoises the data using heat-diffusion, and preserves sample-to-sample affinities when reduced to low dimensions (19).

**Figure 1:**
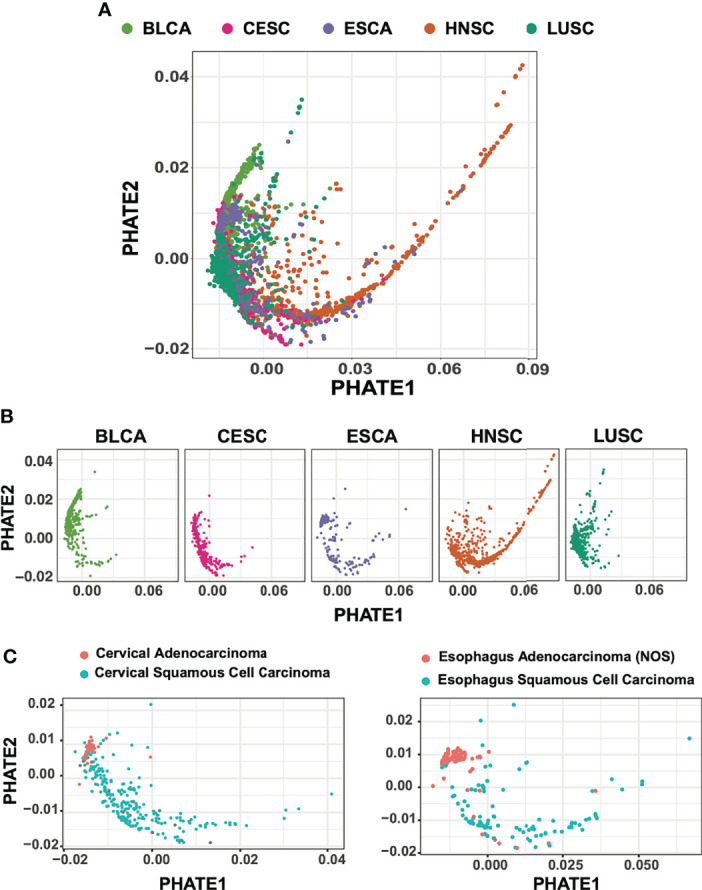
PHATE dimension reductions of 1,925 squamous cell carcinoma subtypes. **(A)** Batch-normalized v2 transcriptomic data of HNSC, LUSC, CESC patient cohorts reduced by PHATE. **(B)** PHATE reductions split by cancer type **(C)** CESC and ESCA adenocarcinomas partition with BLCA urothelial tumors and away from SCC.

For both PHATE and UMAP based analysis, tumor samples across all cohorts were widely distributed (**Figure 1A**, **Supplemental Figures 1A, B**). Also strikingly visible, most clearly in the PHATE reduction output, a large spread of HNSC samples across the PHATE1 axis was observed (**Figure 1B**). We suspect that this is likely due to PHATE’s use of diffusion mapping techniques, and increased valuation of sample-to-sample affinities (33, 34). This large spread of HNSC samples was not entirely surprising given the wide range of tumor heterogeneity and subtypes resulting from different etiological origins.

Although a large proportion of samples included in our analysis were primarily squamous in nature, a subset of tumors from bladder, cervical, and esophageal samples are adenocarcinomas. We next evaluated whether PHATE reduction preserved and separated tumors of varying cellular origins. We found that for both CESC and ESCA samples, adenocarcinoma tumors were tightly associated with other adenocarcinoma tumors, while CESC, ESCA, and HNSC squamous tumors were spread across the PHATE projection (**Figure 1C**). Interestingly, the adenocarcinoma tumors also grouped more closely with the majority of BLCA samples, which are of urothelial transitional-epithelial cell origin, suggesting stronger transcriptional similarities between these two tumor types as compared to tumors of squamous cell origin.

The success of the UMAP and PHATE based analysis prompted us to consider the possibility that this approach can be extended to even larger datasets such as those representing ~11,000 TCGA tumor samples. To this end, we used PHATE successfully to process the bulk RNA-seq data from these tumor samples and as shown in **Supplemental Figure 2**, patient cohorts were finely grouped largely based on tissue of origin. Collectively, these results affirmed the notion that dimension reduction techniques such as UMAP and PHATE provide a robust strategy for visualizing high-dimensional bulk-transcriptomic data from large sample numbers and features, and importantly provide a powerful visualization tool for further characterization.

#### Spectral Cluster Analysis Reveals Hallmarks of Mesenchymal Transition and Provide Robust Clustering of Bulk Tumor Samples

Formation of discrete groups of tumors based on shared biological signal is paramount to cancer subtype analysis, this can indeed guide well informed treatment options and precision medicine. Recently, John et al. developed a strategy to cluster both single and multimodal datasets using spectral clustering, capable of efficiently clustering thousands of input samples with variable distributions (25). We applied this clustering technique to the pan-SCC dataset to assess whether this approach could be extended to large sets of tumor samples.

**Figure 2:**
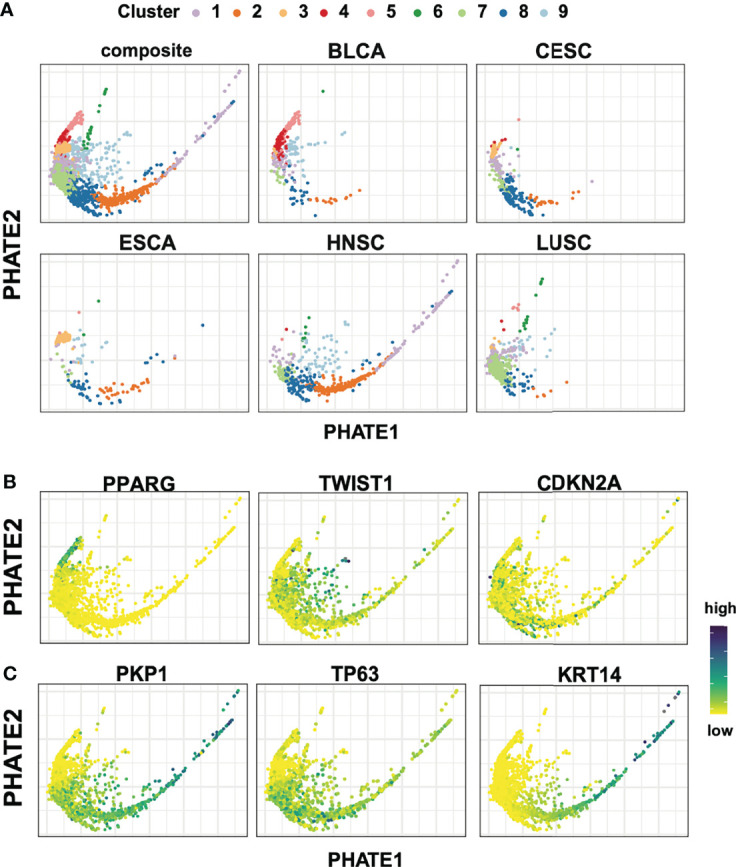
Spectral cluster analysis on 1,925 bulk tumor transcriptomic samples. **(A)** PHATE reductions with spectral clustering results in nine groups of tumors with variable participation for each tumor cohort. **(B)** Expression for *PPARG*, *TWIST1*, *CDKN2A* highlight the heterogeneity across the different cohorts. **(C)** Conserved squamous and epithelial markers genes (*PKP1*, *TP63*, *KRT14*) show a dynamic range of expression in tumors of squamous origin.

Spectral clustering analysis resulted in the partitioning of the 1,925 tumor samples into nine clusters (**Figure 2A**; **Supplemental Figure 3A**). Across these nine clusters, tumors crossed cohort boundaries, though each cluster was predominantly anchored by a specific cohort (**Supplemental Figure 3B, C**). For example, out of the 422 samples in cluster 7, 312 (74%) originated from LUSC, while 89% (84/94) tumors in cluster 5 are BLCA, as marked by urothelial specific marker *PPARG* (**Figure 2B**; **Supplemental Figure 3C**) (35, 36). Cluster formation was also driven by the adenocarcinoma tumors present in our studies. Cluster 3 captured 79.3% (69/87) ESCA adenocarcinomas tumors, and 83.7% (36/43) CESC adenocarcinoma tumors (**Supplemental Figure 3C**). Conversely, clusters 1, 8, and 9 showed significant representation from all five studies, suggesting a strongly conserved squamous cell expression pattern (**Supplemental Figure 3C**).

Next, candidate gene expression profiles were evaluated following differential expression analysis between clusters to further contextualize cluster specific gene expression patterns. Immediately apparent and consistent with previous work were hallmark gene signatures of the Epithelial-Mesenchymal Transition (EMT) characterized by high levels of *TWIST*, *VIM*, *PDGFRA/B*, *SNAI1*, *CYR61* (**Figure 2B**; **Supplemental Figure 4**) (37–40). These signals localize predominantly in cluster 9, and to a lesser extent cluster 1, in agreement with a split in EMT signal previously reported between LUSC and HNSC (**Figure 2B**; **Supplemental Figure 4**) (31). Lastly, as expected, *CDKN2A*, *E2F1*, *E2F2* and *RPA1*, hallmarks of HPV+ tumors, showed enriched expression localized to CESC and a subset of HNSC samples (**Figure 2B**; **Supplemental Figure 4**).

We next sought to understand the relative positioning of tumors in the two-dimensional projections to further extend this analysis. We observed a striking expression gradient in key epithelial associated basal keratin markers such as *KRT14*, as well as in the concerted downregulation of desmosome components including members of the plakophilin family *PKP1* and *PKP3*, and desmosomal cadherin members desmoglein (*DSG1* and *DSG3*), desmocollin (*DSC2* AND *DSC3*), and junction plakoglobin *JUP*. (**Figure 2C**; **Supplemental Figure 4**). In this context, it is worth noting that the loss of desmosome structures has been shown to result in decreased cell-cell adhesion, cancer progression, and increased metastatic potential in head and neck cancers (41). In contrast, *TP63*, a crucial regulatory transcription factor important in development and oncogenesis of epithelial-rich tissues, showed broad and widespread expression across squamous, but not in adenocarcinoma or urothelial tumors (**Figure 2C**) (42–45). This previously undescribed gradient of epithelial gene expression was especially apparent within the HNSC population and to a lesser extent in all five tumor subtypes of squamous origin. The detection of an expression gradient is particularly encouraging, as PHATE’s encoding of local affinities prior to diffusion specifically seek to preserve this latent structure within the data.

These results showcased the strength of dimension reduction techniques, which when paired with spectral clustering can allow for powerful visualization. Importantly, the clustering analysis was performed in an unbiased fashion using the complete transcriptomic profiles of the tumors, without prior feature selection. These results also suggest that this strategy can serve as a generalized framework, and can be applied to diverse tumor populations for in-depth cancer-specific subtyping and molecular analysis.

### Multimodal Dimension Reduction and Classification of HNSC Tumors

A hallmark of head and neck cancer is the immense heterogeneity seen as a result of varying anatomical locations and underlying molecular etiology (31). Due to this fact, and our novel finding of a conserved loss of epithelial and desmosome signatures seen in the pan-SCC analysis, we next performed a robust characterization and subtyping analysis for 514 TCGA HNSC samples. Furthermore, to enhance subtype identification and analysis, we sought to fully integrate multiple data modalities. We posited that molecular classification of the HNSC tumor samples using single data modalities, like RNA-seq or methylation array datasets, might fail to capture the full range of inter tumor heterogeneity and effects of known etiological agents, like HPV+ or NSD1/H3K36 impairment. Towards this end, we applied trimodal spectral clustering analysis paired with PHATE reduction to incorporate RNA-seq, DNA-methylation, and microbiome data that was available for the 514 HNSC samples. For our integrated analysis, each data modality was reduced to a single-view graph, and integrated using cross-view tensor-products, and diffused prior to spectral clustering and dimension reduction using PHATE (25, 26)..

**Figure 3:**
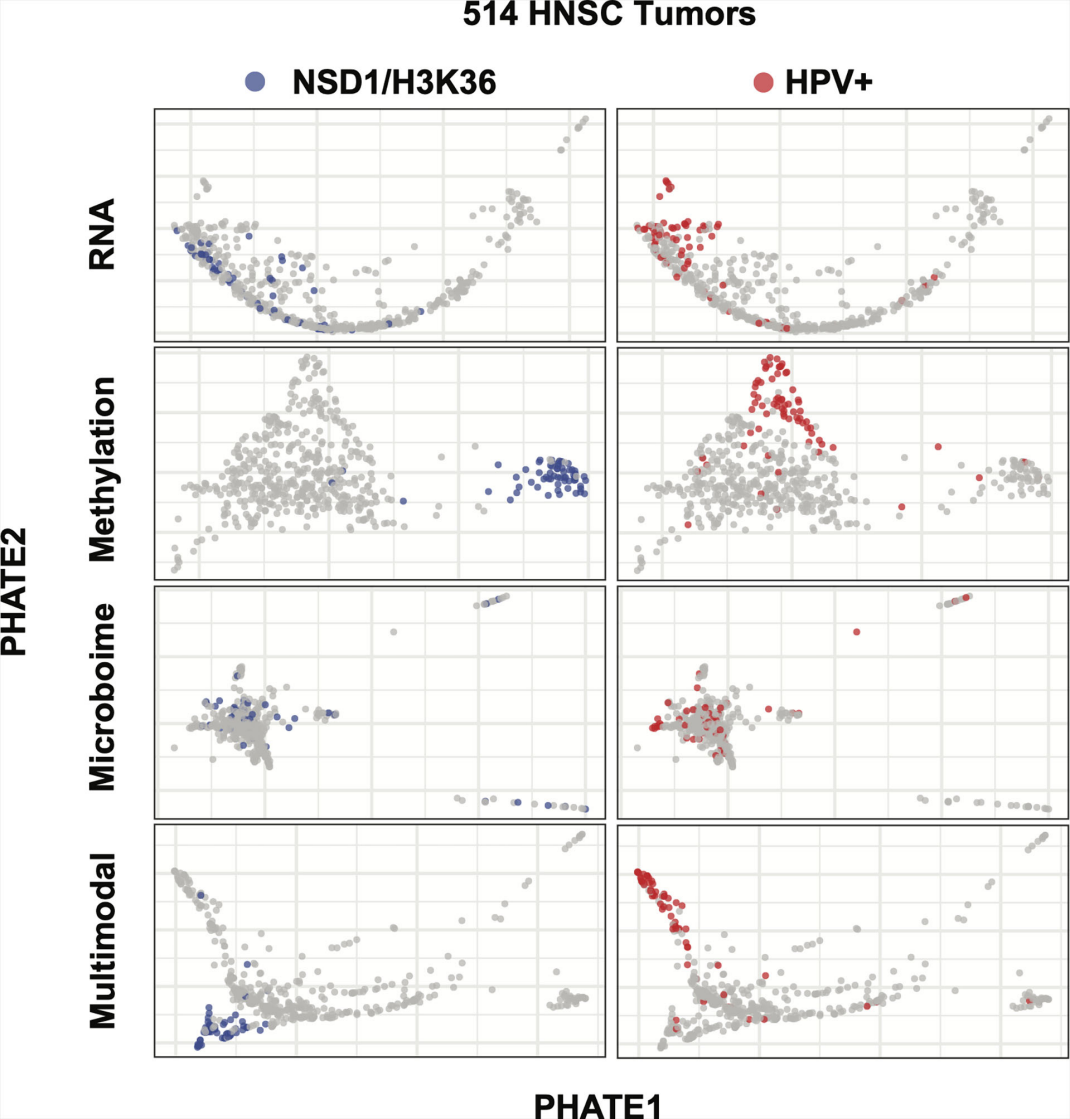
Multimodality dimension reduction as compared to individual data datasets. PHATE dimension reduction on RNA, methylation, or microbiome datasets alone or after being combined into a similarity matrix. Left: NSD1 segmentation was only detectable using the methylation and multimodal reduction. Right: RNA-Seq, methylation and multimodal reductions segment HPV+ tumors.

We tracked samples belonging to two well characterized causes of HNSC, HPV positivity and the methylation-array based signature of impaired H3K36 methylation described by Papillon-Cavanagh et al. (13) to evaluate the effectiveness of multimodal data integration prior to PHATE analysis. PHATE analysis of RNA-seq alone was insufficient in separating the H3K36 impaired tumors, and indeed only showed modest granularity for HPV+ tumors (**Figure 3**). Conversely, PHATE analysis of over 300,00 methylation probe beta values concisely detected impaired H3K36 tumors, as well as HPV+ tumors (**Figure 3**). However, aside from these two cohorts the vast majority of samples remained grouped, suggesting that methylation data alone is insufficient in recapitulating the full range of previously described subtypes. Similarly, using microbiome CPM, PHATE was unable to separate either tumor types (**Figure 3**). Using our fully integrated similarity matrix as input into PHATE, we were able to effectively separate both impaired H3K36 methylation and HPV+ tumor samples, while maintaining the spread of samples previously seen in the RNA-only analysis (**Figure 3**). For these reasons, we chose PHATE analysis of the fully integrated similarity matrix for further multimodal subtype analysis.

#### Multimodal Spectral Clustering of HNSC Tumors to Elucidate Subtype Heterogeneity

**Figure 4:**
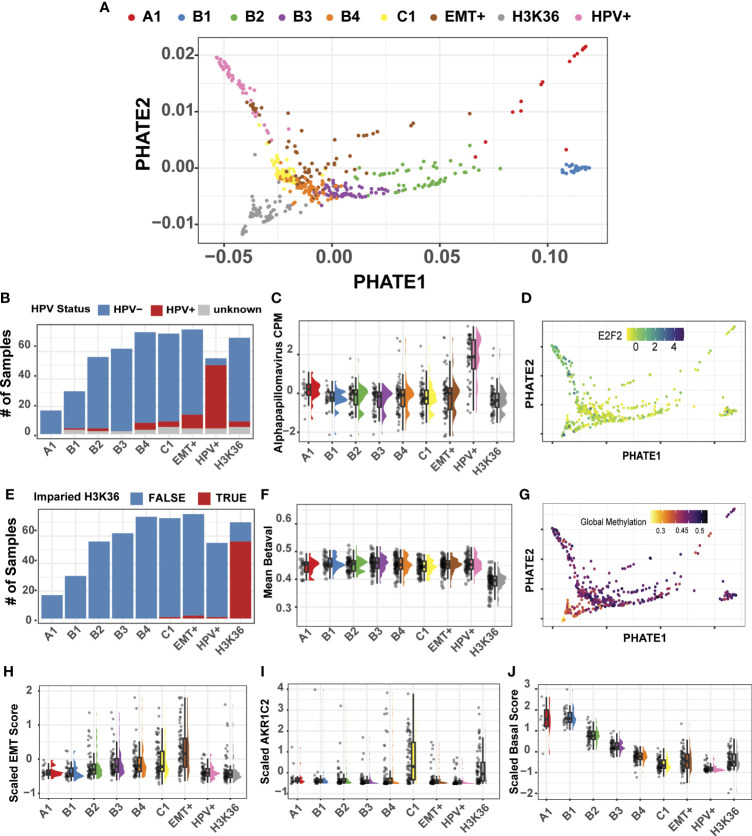
Multimodal clustering and projection of 514 HNSC tumor samples. **(A)** PHATE reduction of the multimodal integrated similarity matrix of transcriptomic, methylation, and microbiome datasets. Spectral clustering generated 9 distinct clusters. **(B)** HPV+ samples predominantly clustered into a single group. **(C)** Alpha papillomavirus counts-per-million from the microbiome dataset are shown for each cluster. **(D)** PHATE projection with expression profile for *E2F2*, HPV+ marker gene. **(E)** H3K36/NSD1 subtype predominantly cluster into a single group. H3K36/NSD1 are defined by Papillon-Cavanagh (13). **(F)** Mean DNA methylation beta values across all probes. **(G)** PHATE projection with global methylation levels. **(H)** Estimate of the Epithelial-Mesenchymal Transition using the Salt et al. EMT score (23). **(I)** *AKR1C2* gene expression marks the classical HNSC subtype. **(J)** Composite score measuring basal marker expression shows a gradient of expression forming from clusters A1-C1.

We next sought to test whether multimodal spectral clustering would partition tumors into biologically meaningful clusters using our multimodal PHATE projection as the basis for visualization. Spectral clustering analysis of 514 HNSC tumor samples generated nine clusters, ranging from 17 samples (cluster A1) to 76 samples (EMT+) with a median sample-per-group count of 62 (**Figure 4A**; **Supplemental Figure 5**). Previous analysis of HNSC had revealed four predominant subtypes—Basal, Classical, Mesenchymal, and Atypical/HPV+ (12, 46–49). We next evaluated if our clustering was rooted in the four-subtype system, or if multimodal inputs provided a more granular partitioning of the HNSC patient cohort that more accurately mirrored the multiple etiologies and the underlying distinct transcriptomic and DNA methylation status of the tumors.

##### HPV+ and Impaired H3K36 HNSC Subtypes Partition Into Discrete Clusters

Using available clinical metadata, we first evaluated the HNSC HPV subtype, revealing that HPV+ tumor samples generated one predominant group (46/72, 64%) located at the left extreme of the PHATE1 axis (**Figures 4A, B**). Recent additions of microbiome measurements for TCGA patient cohorts enabled us to include a third modality for our analysis (24). As expected, the top genus, Alpha papillomavirus was upregulated in the HPV+ cluster (**Figure 4C**). Indeed, visualization of a well-established HPV+ marker, E2F2, confirmed the HPV+ group positioning (**Figure 4D**) (47).

Encouraged by the accurate clustering of HPV+ tumors, and their defining microbiome and transcriptional signatures, we next sought to evaluate if our multimodal clustering approach could efficiently segregate the subset of HNSC tumors that were previously identified by methylation-based signature of NSD1/H3K36 impairment (13). Since the previous analysis was performed using only the top 1,000 variable probes, we employed the complete set of methylation arrays, with over 300,000 probe beta-values for our multimodal analysis. Indeed, multimodal clustering was sensitive to this methylation-based signature, grouping 56 out of the 60 (93%) tumor samples into a single cluster harboring a mutation in NSD1/H3K36 (**Figures 4A, E**). Our result demonstrated that no-prior sub setting of methylation probes was required and that a complete representation of the methylation dataset in downstream analyses is feasible, if needed. Also consistent with the results from Papillion-Cavanagh et al., was the global hypomethylated state in the NSD1/H3K36 tumors (**Figures 4F, G**). Beyond the advantage of the inclusion of large-scale data points, our approach also enabled us to examine data at probe-specific resolution. For example, two probes previously reported as differentially methylated at *TP63* transcriptional start sites were cluster specific, cg16764781 and cg06520450 (31). Our analysis reveals that the probe cg16764781 is hypomethylated in the HPV+ group, and is largely specific to that subtype (**Supplemental Figure 6**). Taken together, integration of these datasets allowed for precise evaluation of differential expression, microbiome abundances, and methylation statuses both at the global and individual probe level.

##### Traditional Markers of Epithelial-Mesenchymal Transition, High Tumor Mutational Burden, and an Epithelial Gradient of Expression Defines Specific HNSC Clusters

To further characterize the remaining clusters, we focused on mesenchymal markers and utilized a gene-expression based on the Epithelial-Mesenchymal Transition (EMT) score as previously described (23). The estimated EMT score increased from clusters A1 to our labelled EMT+ cluster, with the highest expression levels of known mesenchymal markers in EMT+ as compared to all other clusters (**Figure 4H**; **Supplemental Figure 7**). Our EMT+ subtype exhibit elevated markers *VIM*, *SNAI1*, and *ZEB1*, consistent with our pan-cancer analysis and with known molecular features of epithelial-mesenchymal transition.

Upon resolving the identity of the remaining clusters, we found cluster C1 to be enriched for markers associated with the classical HNSC subtype, such as *ALDH3A1*, *AKR1C1*, and *ARK1C3* (**Figure 4I**) (12). *AKR1C1/3* are up regulated in response to xenobiotic substances like cigarette smoke, a primary HNSC cancer etiological agent (46). C1 tumors also harbor high mutational burden, as well as loss of 1P, and 4P, and amplification of chr3 q26.33-q27.1 in >40% of the samples (**Supplemental Figure 8**). This amplified genomic segment referred to as the q26.33 OncCassett, includes the gene for *SOX2*, a well characterized transcription factor involved in maintaining stem-like phenotypes in HNSC (**Supplemental Figure 9**) (46, 48, 50, 51). Lastly, we found that cluster A1 exhibited variable differences in transcriptional and microbiome signatures and is comprised of only 17 tumor samples. The variability of A1 cluster, we suspect is likely influenced by its low sample number.

In contrast to the EMT+, impaired NSD1/H3K36, and HPV+ tumors, which formed relatively discrete clusters, evaluation of epithelial markers using a composite basal score of epithelial markers, displayed decreasing levels across four clusters, labelled B1, B2, B3, and B4 (**Figure 4J**). This expression gradient was consistent with the HNSC spread seen in the pan-cancer analysis, with decreased expression in the keratin and desmosomal family of genes as shown in our pan-SCC analysis (**Supplemental Figure 4**). Taken together, our multimodal spectral cluster analysis of the 514 HNSC tumors using methylation, transcriptomic, and microbiome-based signatures, partitioned samples into biologically meaningful cohorts, in large agreement with previously established subtyping. However, the existence of a well-defined gradient of gene expression pattern across the basal subtype was a novel observation and thus became the focus of our follow-up analyses.

##### Basal Subtype Analysis Confirm Loss Of Epithelial and Desmosomal Gene Expression, Changes in Methylation Patterns, and Altered Tumor Microbiome

**Figure 5:**
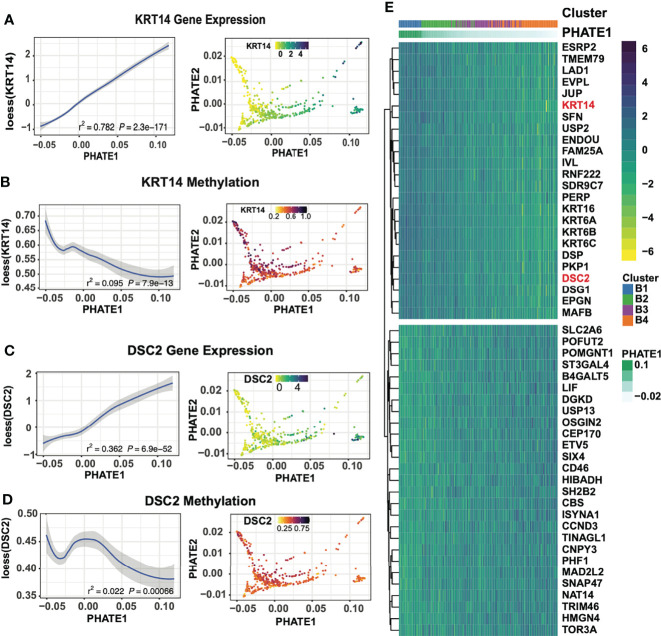
Basal-focused subgroup analysis exploring transcriptional expression gradients. **(A)** *KRT14* gene expression across the PHATE1 axis using loess curve smoothing, and on the PHATE projection. **(B)** DNA methylation at *KRT14* associated probes across the PHATE1 axis using loess curve smoothing, and on the PHATE projection. **(C)** *DSC2* gene expression across the PHATE1 axis using loess curve smoothing, and on the PHATE projection. **(D)** DNA methylation at *DSC2* associated probes across the PHATE1 axis using loess curve smoothing, and on the PHATE projection. **(E)** Heatmap of the top 30 DEGs between cluster B1 and B4 show shift of epithelial and desmosome gene expression, ordered by the PHATE1 axis.

To further characterize the basal subclusters, we first identified key transcriptomic features through pairwise differential expression between basal subgroups. Top differentially expressed genes exhibit a decreasing gradient of expression along the PHATE1 axis of several Keratin and desmosome genes including *KRT14*, *DSC2*, *KRT5*, *PKP1*, *DSP*, *JUP* as well as *SIX4*, *RUNX2*, and *S100A8* (**Figures 5A, C, E**; **Supplemental Table 1**). We suspect that this conserved expression gradient prevalent across numerous genes is likely the driver of the PHATE1 axis spread, similar to the gradient formed in the pan-SCC analysis. This is consistent with diffusion mapping algorithms which encoded both local sample-to-sample affinities, as well as global structure between individual samples (33, 34). Indeed, these tumors exhibited gradients in all three data modalities. For example, *KRT14* and *DSC2* associated probes increased in methylation levels across the PHATE1 axis, and microbiome levels as seen across numerous genus (**Figures 5B, D**; **Supplemental Figure 10**). Strikingly, within the microbiome data distinct patterns of genus abundance are detected within our clusters, with Lactobacillus, Manheimia, Prosthecomicrobium, and Microvirga showing similar gradients across the basal clusters (**Supplemental Figure 10**). Extending this analysis by overlaying tumor hypoxia estimates using the Ragnum Hypoxic Score also resulted in a gradient along the basal spectrum, with highest hypoxic levels in the B4 and C1 clusters (**Supplemental Figure 11**). Furthermore, Ingenuity pathway analysis of up and down regulated genes between the two extremes of the basal spectrum showed transition from normal epithelial differentiation in B1 cluster to more invasive and aggressive SCC in B4 (including activation of proliferation, migration, and invasion ontologies) (**Supplemental Figure 12, Supplemental Table 2**) that correlated with increasing tumor grade (**Supplemental Figure 13**).

##### Immune and Stromal Cell Influence and Partial-EMT

**Figure 6:**
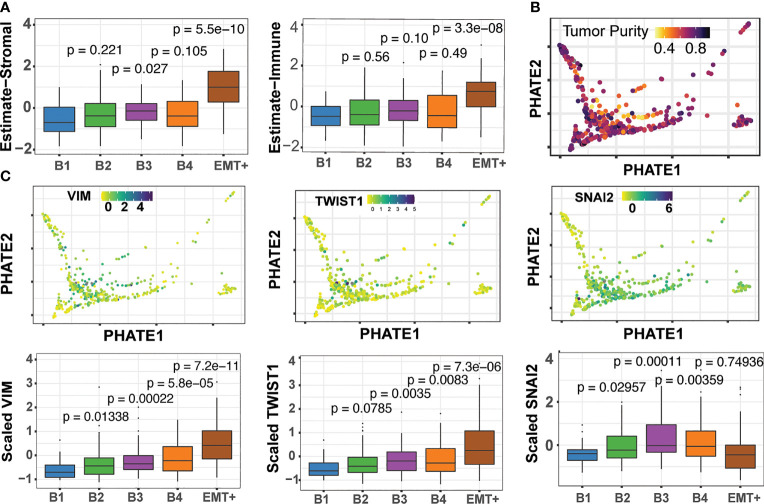
The Epithelial-mesenchymal transition and detection of pEMT. **(A)** ESTIMATE stromal and immune scores across the B1 to B4 and EMT+ clusters (56). Pairwise Wilcoxon tests for each cluster compared back to B1, with associated p-values **(B)** Consensus tumor purity score derived by Aran et al. (55) **(C)** Expression of the EMT marker genes across basal clusters.

Through the use of single-cell sequencing data, the transcriptomic signatures that underlie EMT have been deconvoluted from tumors with high immune and stromal influences (52–54). We applied the ESTIMATE algorithm, which measures the immune and stromal content of tumor samples to evaluate whether any of our clusters showed high immune and stromal cell influence. ESTIMATE showed our EMT+ cluster had increased scores for both immune and stromal signatures, as well as a decrease in tumor purity as estimated by Aran et al. (55) (**Figures 6A, B**). This, we suspect, is likely the source of the separation of the EMT+ cluster from the B1-B4 basal groups (**Figure 6A**). We found *TWIST1* and *VIM* to be increased along the basal trajectory, while *SNAI2* showed higher expression in B3 and B4, while not significantly different in the EMT group (**Figure 6B**). This pattern of *SNAI2* “peaking” expression in cluster B3 suggests *SNAI2* occupies an intermediate stage of the EMT process as reported previously (57). Similarly, *ITGA5* was found to be enriched in both the B3/B4 and EMT+ clusters, consistent with the oncogenic integrin signaling between tumor microenvironment and the recently described EMT-like tumor specific keratinocyte (TSK) cell populations in cutaneous SCC (**Supplemental Figure 14**) (54). Lastly, *LAMC2*, *S100A8*, and *KLK11* expression patterns were in agreement with the partial-EMT state of HNSC as described by Puram et al. (**Supplemental Figure 14**) (53, 54). The robust detection of the nuanced partial-EMT states in bulk-tumors further highlight the efficacy of our multimodal analysis strategy.

##### Multimodal Clustering Segments Tumor Groups With Variable Survival Outcomes

**Figure 7:**
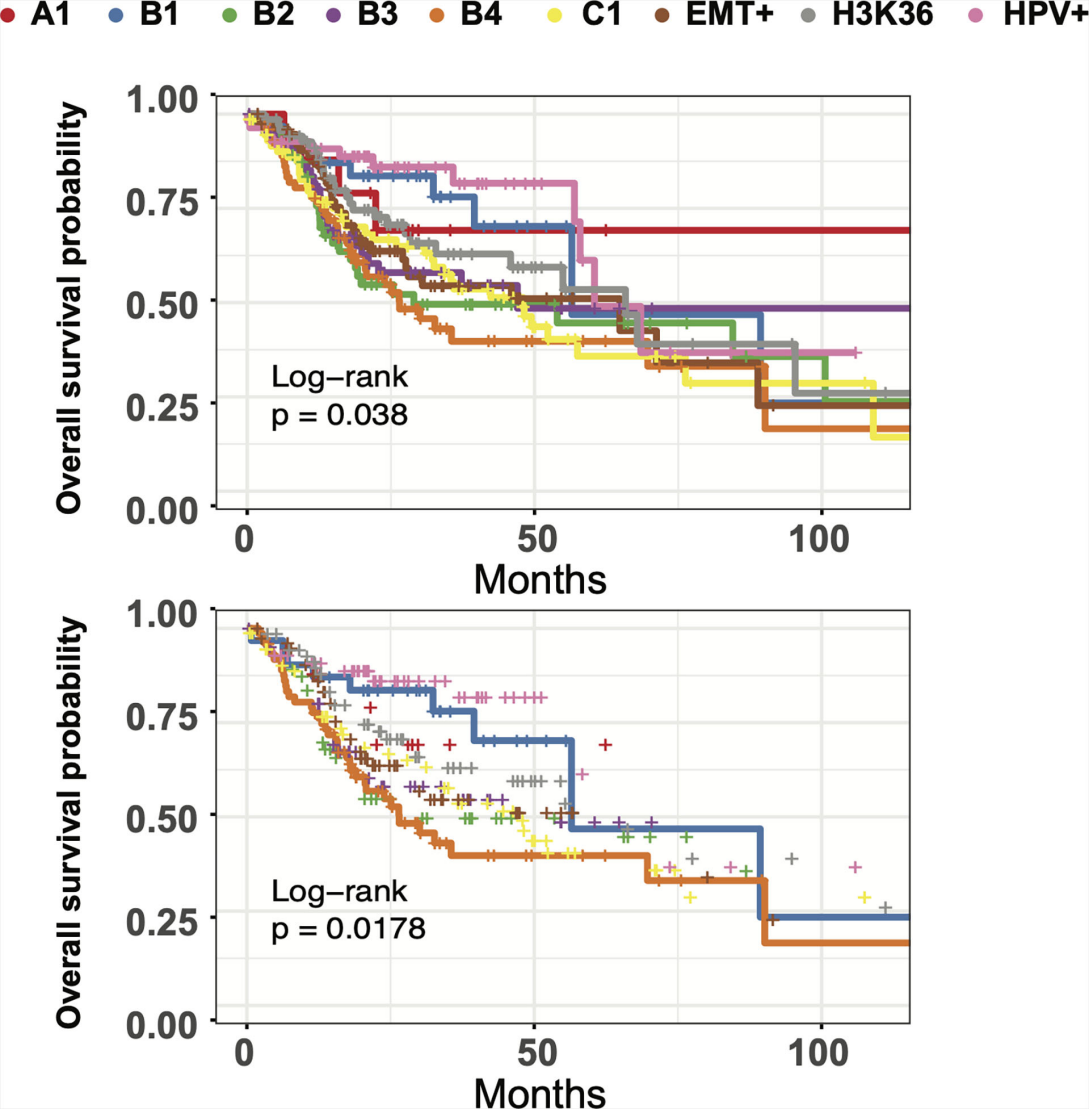
HNSC subgroups have differing times to median survival and long-term survival. Top: Kaplan-Meier curve analysis of all 9 multimodal clusters overall survival using the Log Rank Test with pval = 0.0380 (q=0.076). Bottom: Kaplan-Meier curve analysis of B1 and B4 clusters using the Log Rank Test with pval = 0.0178 (q=0.0308).

We next sought to evaluate clinical outcome for our HNSC clusters. Kaplan-Meier survival curve analysis illustrated stark differences in 5-year overall-survival rates, indicating clinical ramifications for the underlying heterogeneity seen in our multimodal spectral clusters (**Figure 7**). HPV+ samples, EMT+, and NSD1/H3K36 groups were associated with the best median survival of ~62 months, while cluster B4 had the lowest among the four basal clusters. Indeed, tumors grouped in B4 had the worst median survival of ~26.5 months, across all 9 groups. The median survival outcomes for the B1 group were nearly twice as long at a median of 56.48 months. Lastly, cluster B1 exhibits elevated expression of *CYSRT1*, a gene previously identified as positively correlated to overall-survival, consistent with Kaplan-Meier curve analysis (**Supplemental Figure 15**) (58). The pairing of two flexible analysis strategies, PHATE dimension reduction and multimodal spectral clustering, aided in us in identifying subgroup heterogeneity linked with survival outcomes in HNSC, and allowed for detailed interrogation of underlying methylation, transcriptional, and microbiome patterns through unbiased multimodal data integration.

## Discussion

Large scale and integrated analysis of tumors, often based on multiple data modalities has revealed immense heterogeneity for most cancers. What has become quite clear from such studies is that cancers originating from the same human tissue or organ can dramatically differ in their etiology, pathology and the underlying genomic and epigenomic alterations. Precise tumor classification and identification of cancer subtypes based on underlying molecular signatures is thus an important step towards better understanding of the disease and importantly, for targeted therapy. This has led to recent studies such as the PanCancer Atlas integrative analysis, which identified 28 distinct molecular subtypes arising from the 33 different tumor types (21). Indeed, the unsupervised consensus clustering of tumor profiles performed across multiple TCGA genome-wide platforms including DNA methylation and RNA sequencing in the aforementioned study have reaffirmed the striking diversity of tumors and offered new insights into their molecular taxonomy. However, despite these advances, it is likely that there exist fine-grained tumor subtypes that are yet to be discovered. Such discovery would benefit from integrated application of new analytical and visualization tools to better parse the diverse sets of multi-dimensional data generated by high-throughput technologies.

Towards this end, several powerful strategies have been developed to interrogate multi-omic datasets (59). Examples of such strategies include low rank approximation based multi-omics data clustering (LRAcluster), which relied on linearly concatenate multi-omic profiles prior to probabilistic modeling and clustering of a latent subspace (60). This dimension reduction strategy allowed for integrative clustering of large-scale cancer multi-omics data. Other examples, including perturbation clustering for data integration and disease subtyping (PINS) and cluster-of-cluster assignments (COCA), which cluster each data view separately before they are integrated through consensus clustering strategies (61, 62). Similarly, statistical approaches using Bayesian statistics like canonical correlation analysis (CCA), iClusterBayes, BCC, are also capable of multi-modal analysis, with the added benefit of providing the probability of a sample belonging to a given cluster (63–66).

As an alternative to such integration algorithms, similarity-based strategies rely on first computing sample-to-sample measures of similarity or differences, prior to integration. This initial computation lends itself well to omic-based datasets of variable distributions (59). Recently, MoGCN has parlayed the advantage of autoencoders paired with neural networks for cancer subtype classification and analysis of breast invasive carcinoma (BRCA) samples (67). Indeed, similarity-based integration paired with spectral clustering techniques have provided an efficient method to handle complex genomic datasets of varying distributions (25, 26). Building upon these studies, here we present a computationally efficient analysis framework using similarity-based integration that allows for inter-cluster inspection and visualization at a much granular resolution.

We showcase the benefit of an integrated approach that relies on multimodality spectral clustering paired with dimension reduction techniques on large and complementary cancer genomic datasets. Indeed, we demonstrate the flexibility and performance of these algorithms, which in a seamless and robust fashion can handle datasets of varying size such as the pan-Cancer, pan-SCC and the HNSC patient cohorts from TCGA. Additionally, our strategy overcomes the limitation of traditional clustering approaches, which operate on single-view representations of a given sample set, and are designed to assign community participation based on a distance heuristic (68). This is particularly relevant for high dimensional data, like those analyzed in this study. Our results also suggest that the traditional classification approach of organizing tumor samples into discrete bins prior to calculating an average tumor profile based on a single mode of data offers only a static and limited view. Cancer progression is intimately associated with dynamic molecular changes in the molecular gene expression, DNA methylation states, and other modalities that are often transient in nature. We show that powerful dimension reduction techniques such as PHATE offer a better understanding of these transitions by ordering individual samples along these trajectories thus generating a non-discrete representation of the heterogeneity that is deeply inherent to most tumors.

We sought to probe the efficacy of dimension reduction and multi-view clustering strategies on HNSC tumors given that these represent distinct anatomical locations, diverse molecular mechanisms of carcinogenesis and highly heterogeneous tumor microenvironment. Unsurprisingly, patients with HNSC tumors have varying clinical responses among the 3-5 molecularly defined subtypes that have been primarily clustered anchored on single-data-view representations (69, 70). For our approach, we utilized integrated methylome, transcriptome, and microbiome signatures on TCGA HNSC tumors using recent advances in multi-graph integration using three data modalities. The nine clusters that were identified in our analysis included known HNSC subtypes such as the HPV+ and NSD1/H3K36 impaired groups, aided by inclusion of the tumor-specific microbiome, and DNA-methylation status, respectively. However, unlike previous reports, we found the basal subtype to be quite complex with four well-partitioned clusters that reflected a gradient of gene expression typified on one end by keratin enriched (B1) and mesenchymal markers (B4) on the other. These clusters also exhibited gradients in DNA methylation states and associated microbiome (Lactobacillus, Wolbachia, and Mannheimia among others) levels, further highlighting the power of the multimodal analysis. The incorporation of the microbiome state in our analysis is particularly interesting and worth further investigation because it is becoming clear that oral and gut microbiome are associated with HNSC development, progression, treatment and its potential side effects (71).

Several additional observations from our analysis of the HNSC tumors are note-worthy. First, by preserving global sample-to-sample relationships while ordering tumors by local affinities, PHATE highlighted the dynamic range of expression along the Epithelial-Mesenchymal transition (EMT) state in HNSC tumors. Furthermore. our study reaffirmed the evolving notion that tumor ecosystems often exhibit a continuum of meta-stable, intermediary pEMT (or hybrid) states between the epithelial and mesenchymal poles (72, 73). Second, clear segmentation of tumor samples characterized by heterogeneous cell populations and low tumor purity, such as stromal and immune cells, demonstrate the robustness and sensitivity of dimension reduction algorithms. We posit that unlike in the case of principal component analysis where the first two to three dimensions are driven by global variance, PHATE allowed us to model a broader and accurate representation of the multi-cellular tumor ecosystem. Indeed, this information is often lost using commonly used hierarchical clustering strategies which rely on pre-selected gene lists or only subsets identified *a priori* by principal component analysis.

Current treatment options for HNSC primarily consist of surgery, radiation and chemotherapy, administered in single or multi-modality regimens. However, these treatments still leave room for substantial improvement in efficacy and importantly does not consider molecularly defined subsets. Furthermore, approval of immunotherapy drugs such pembrolizumab and nivolumab for adjuvant treatment of recurrent and metastatic, or advanced unresectable HNSC, underscore the urgent need for research into tumor heterogeneity to better identify patients that are likely to be responders (74, 75). Given the complex link between immunomodulation and tumor microenvironment, a more robust and accurate classification strategy will be beneficial (76). Although we used HNSC to test our multimodal strategy, our studies provide a generalizable framework that can be applied to any cancer or other diseases. In addition, our approach can easily incorporate additional genomics data beyond methylation, transcriptome, and the microbiome. Finally, it is also important to consider novel data imputation strategies to maintain robust sample numbers in the event that not all data modalities are collected and available for each patient. Evolving techniques such as generative adversarial neural networks, among other machine learning strategies, can provide accurate predictions of both transcriptomic and methylation states that could be incorporated for multimodal clustering (77–79). Development of rapid and scalable multiomics data integration and mining strategies will continue to enable better modeling of the inherent heterogeneity of tumors and offer molecular insights into their complex and granular landscape.

## Data Availability Statement

Publicly available datasets were analyzed in this study. This data can be found here: https://www.cbioportal.org/comparison/overlap?comparisonId=60804602e4b0242bd5d4984c.

## Author Contributions

JB conceptualized the project, acquired and analyzed data, prepared the figures, and wrote the manuscript. NN provided funding and participated in the manuscript review and editing. MB and SS supervised the project, wrote and edited the manuscript, and provided funding. All authors contributed to the article and approved the submitted version.

## Funding

This project was supported by grants from the National Institutes of Health (R01GM132199 to MJB, and R01AR073226 to SS) and Community Foundation for Greater Buffalo to MB, SS, and NN.

## Conflict of Interest

The authors declare that the research was conducted in the absence of any commercial or financial relationships that could be construed as a potential conflict of interest.

## Publisher’s Note

All claims expressed in this article are solely those of the authors and do not necessarily represent those of their affiliated organizations, or those of the publisher, the editors and the reviewers. Any product that may be evaluated in this article, or claim that may be made by its manufacturer, is not guaranteed or endorsed by the publisher.
