# Supplementary material for: Multimodal Dimension Reduction and Subtype Classification of Head and Neck Squamous Cell Tumors
Source: Front Oncol. 2022 Jul 13;12:892207. doi: 10.3389/fonc.2022.892207 (PMC9326399; doi:10.3389/fonc.2022.892207)
Supplement: Supplementary file 1 [file Presentation_1.zip › Supplemental Figures_Reviews.docx]

# Supplemental Figures

# Multimodal dimension reduction and subtype classification of head and neck squamous cell tumors.

Jonathan E. Bard^1,2^, Norma J. Nowak^1,2^, Michael J. Buck^1,3*^, Satrajit Sinha^1*^

^1^ Department of Biochemistry, Jacobs School of Medicine and Biomedical Sciences,

State University of New York at Buffalo, Buffalo, New York, USA.

^2^ Genomics and Bioinformatics Core, Jacobs School of Medicine and Biomedical Sciences, State University of New York at Buffalo, Buffalo, New York, USA.

^3^ Department of Biomedical Informatics, Jacobs School of Medicine and Biomedical Sciences, State University of New York at Buffalo, Buffalo, New York, USA.

**Supplemental Fig 1. UMAP dimension reductions of 1925 squamous cell carcinoma subtypes.** (A) Batch-normalized v2 transcriptomic data of HNSC, LUSC, CESC, patient cohorts reduced by UMAP. (B) UMAP reductions faceted by cancer type.

**Supplemental Fig 2. PHATE dimension reductions of 11070 TCGA Pan Cancer Samples.** (A) Batch-normalized v2 transcriptomic data of the full TCGA Pan Cancer dataset.

**Supplemental Fig 3. UMAP dimension reductions and spectral cluster analysis on 1925 bulk tumor transcriptomic samples.** (A) UMAP reductions with spectral clustering results in nine groups of tumors with variable participation for each tumor cohort. (B). Percentage of tumor cohort assigned to each spectral cluster. (C). Participation of tumor cohort in each cluster.


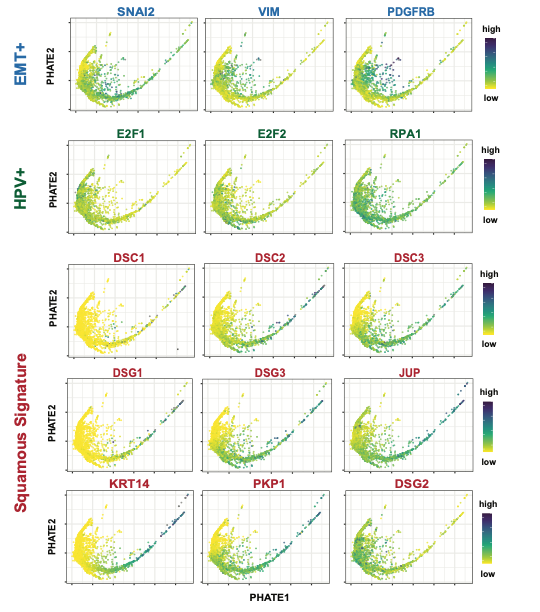


**Supplemental Fig 4. Expression profiles of select markers in 1925 bulk tumor transcriptomic samples in PHATE dimension reduction.** (Blue) Expression for SNAI2, VIM, PDGFRB highlight nuanced heterogeneity across the different cohorts. (Green) Hallmark HPV+ expression for E2F1, E2F2, and RPA1. (Red) Conserved squamous, epithelial, and desmosome component genes (DSC1, DSC2, DSC3, DSG1, DSG3, JUP, KRT14, PKP1, DSG2) highlight a dynamic range of expression localized to tumors with squamous origin, with a strong gradient in HNSC tumors.

**Supplemental Fig 5. Sample assignments to each spectral cluster following multimodal integration.** A1 (n=17), B1 (n=31), B2 (n=56), B3 (n=62), B4 (n=74), C1 (n=73), EMT+ (n=76), HPV+ (n=55), H3K36 (n=70)


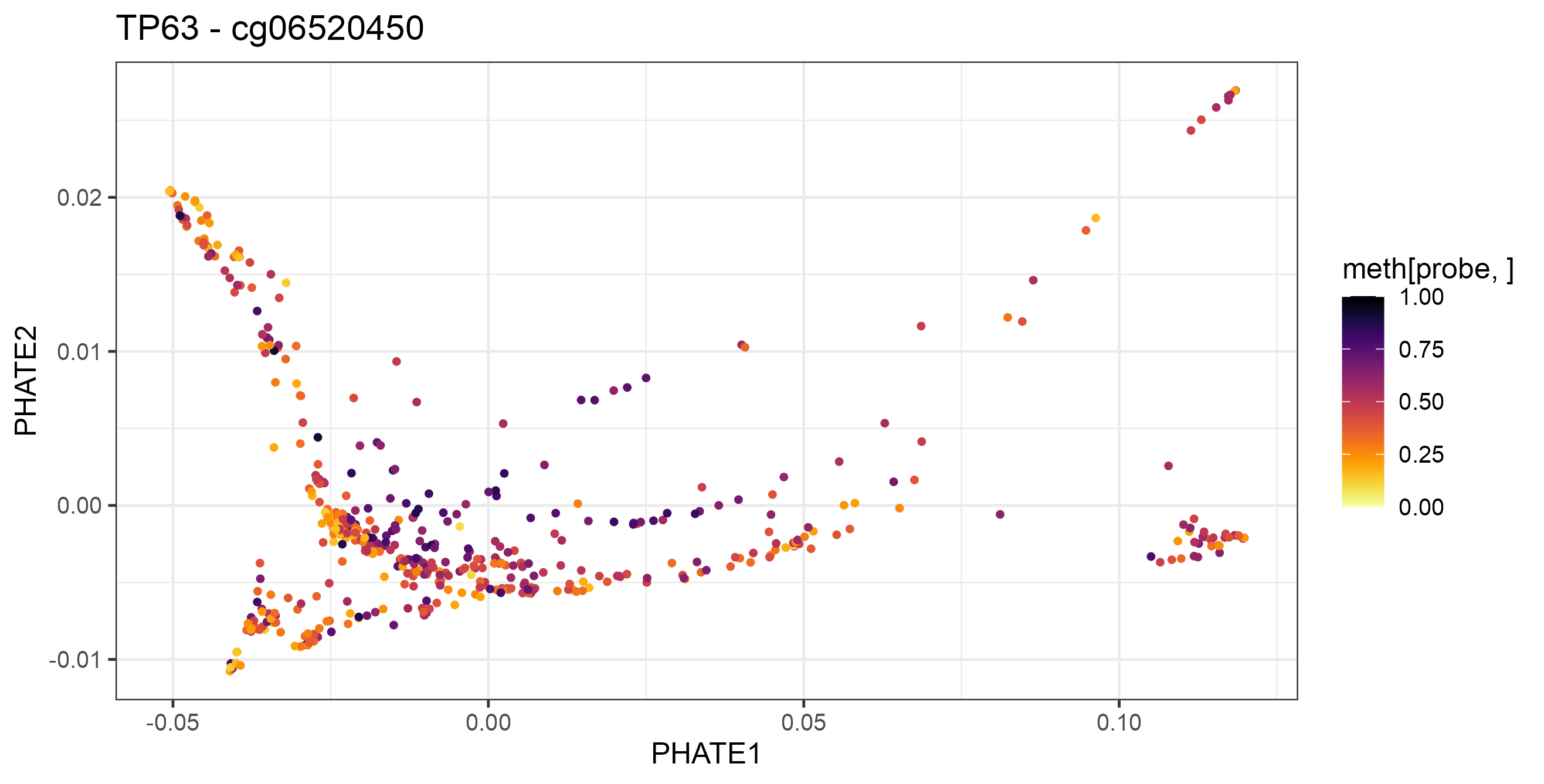

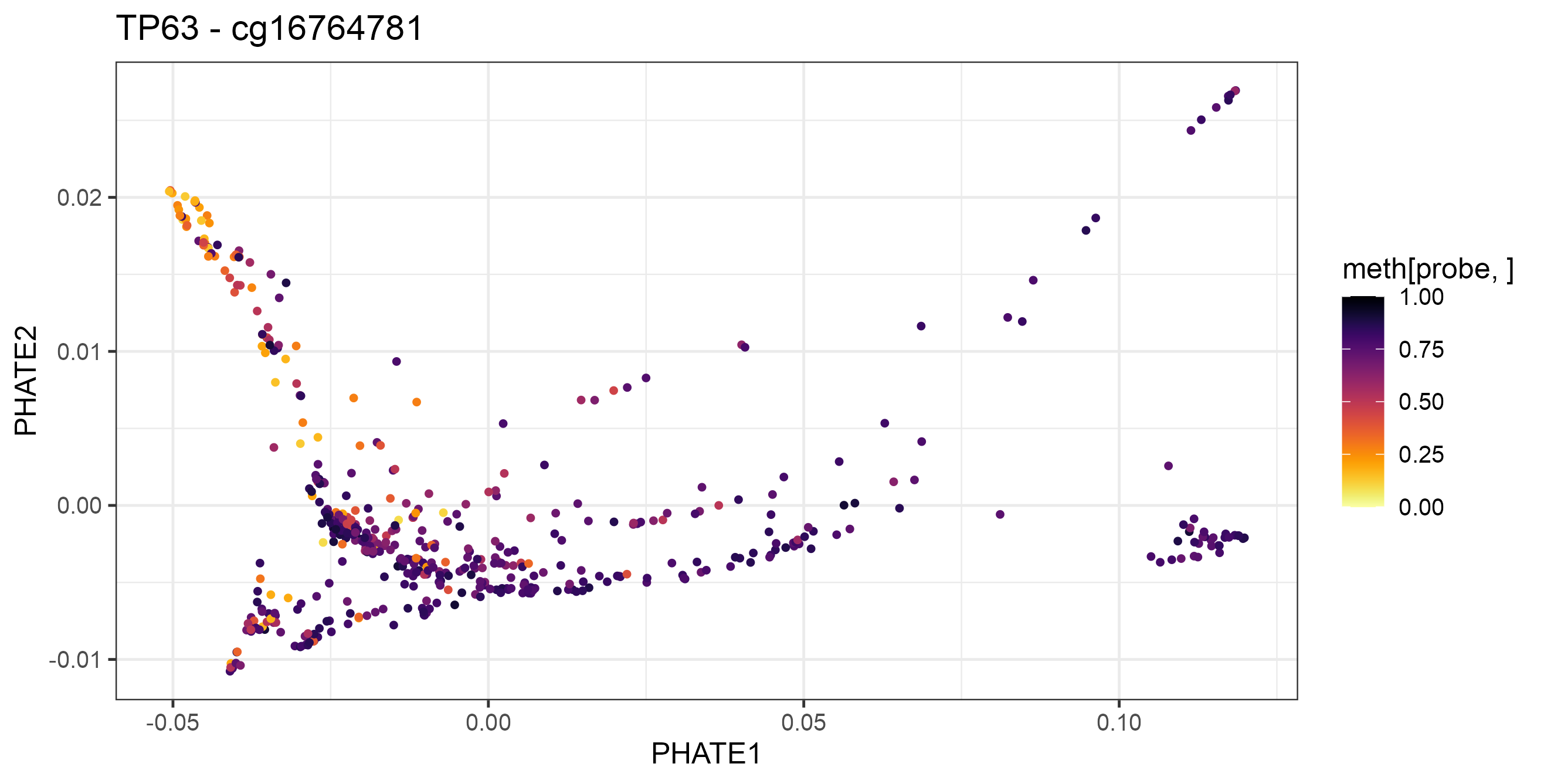

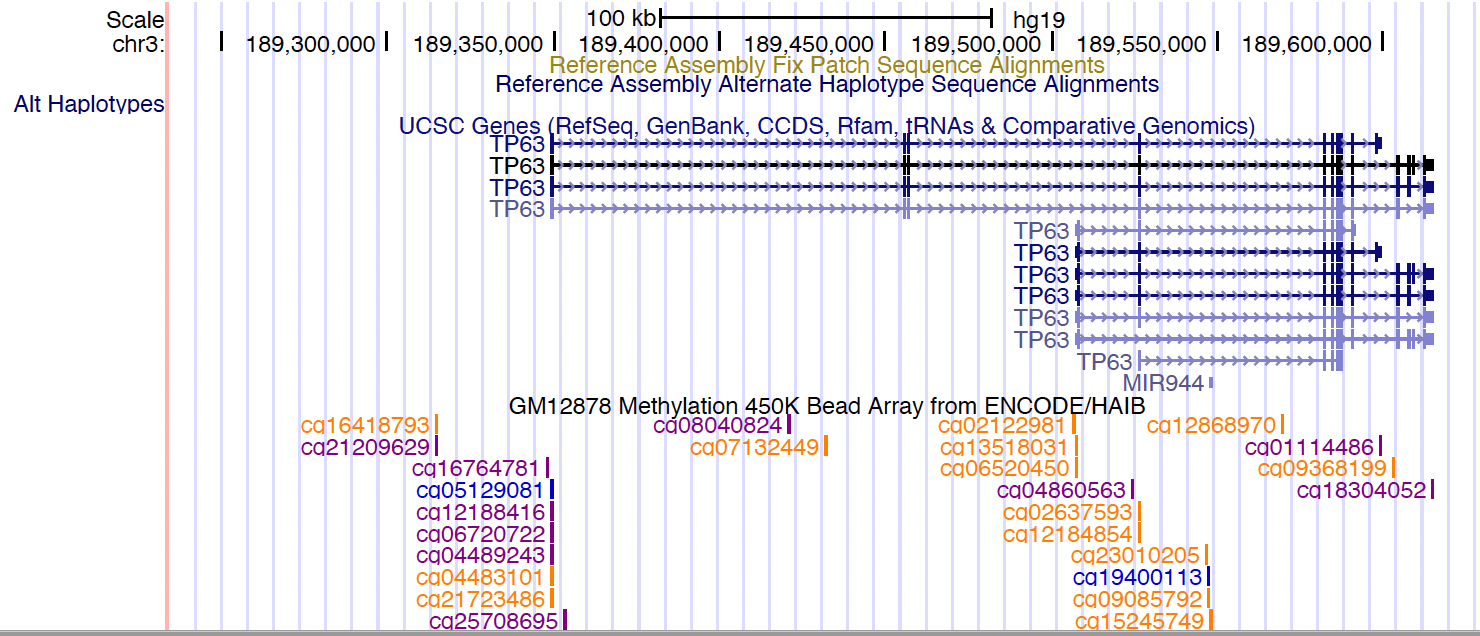


**Supplemental Fig 6. Cluster specific methylation patterns at TP63 isoform promoters.** Left- TA-TP63 promoter marked by probe cg16764781 highlight cluster-dependent hypomethylation within the HPV+ tumor samples. Right- ΔN-TP63 probe cg06520450 show dynamic range of methylation levels between samples of different clusters.


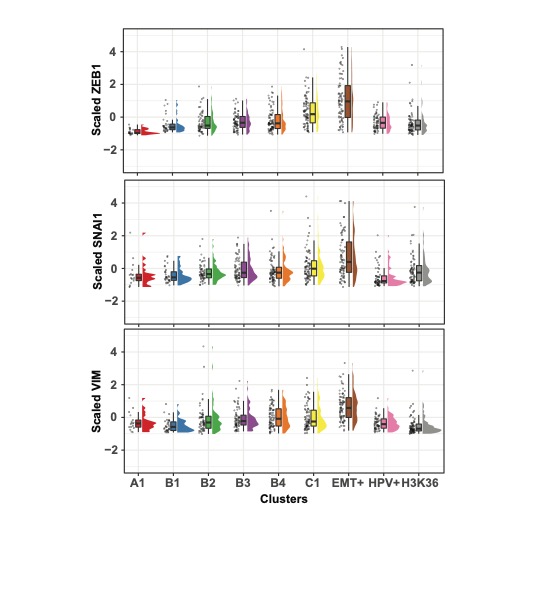


**Supplemental Fig 7. Hallmark expression profiles of Epithelial-Mesenchymal transition across spectral HNSC clusters.** Scaled expression of ZEB1, SNAI1, VIM.


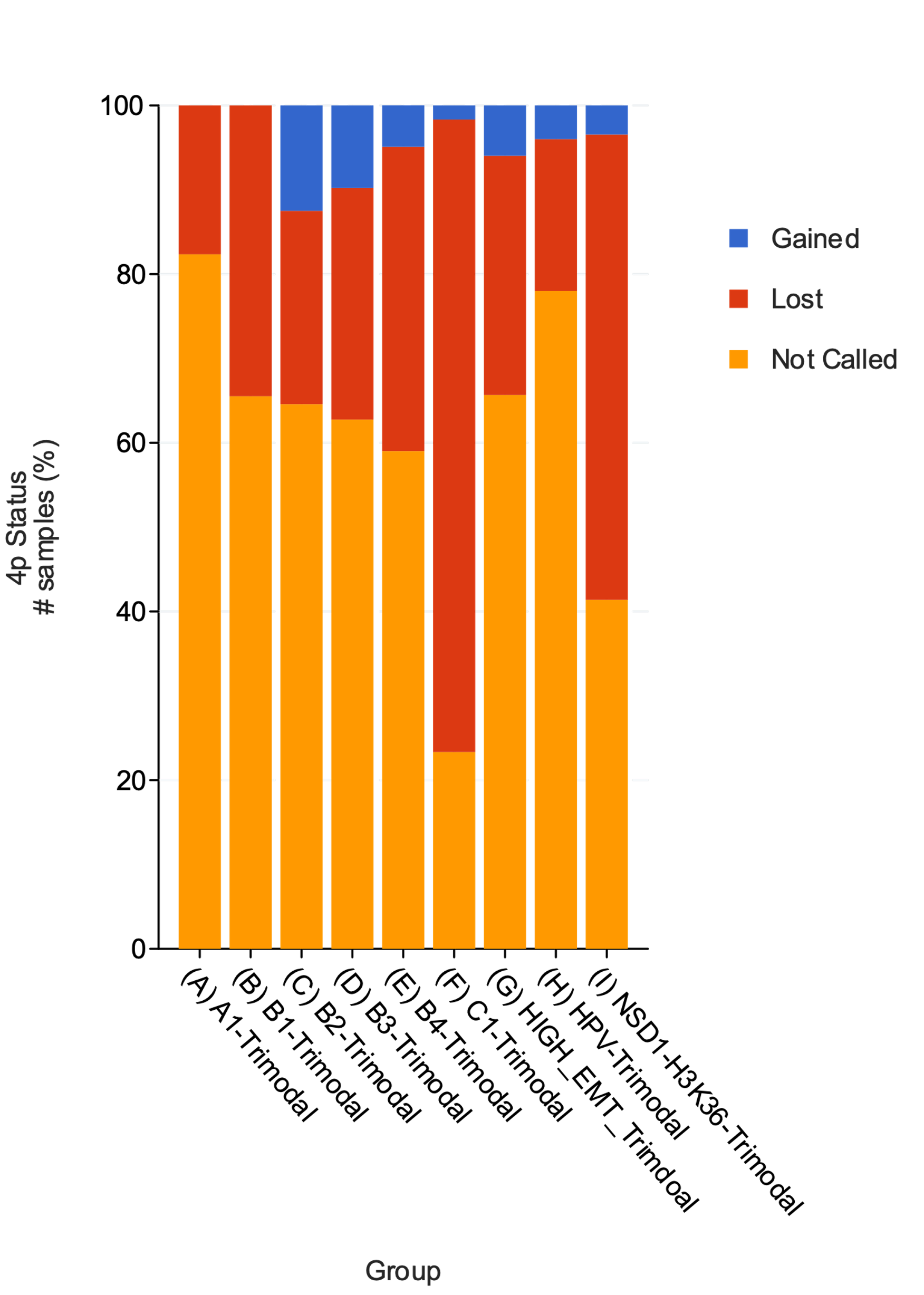

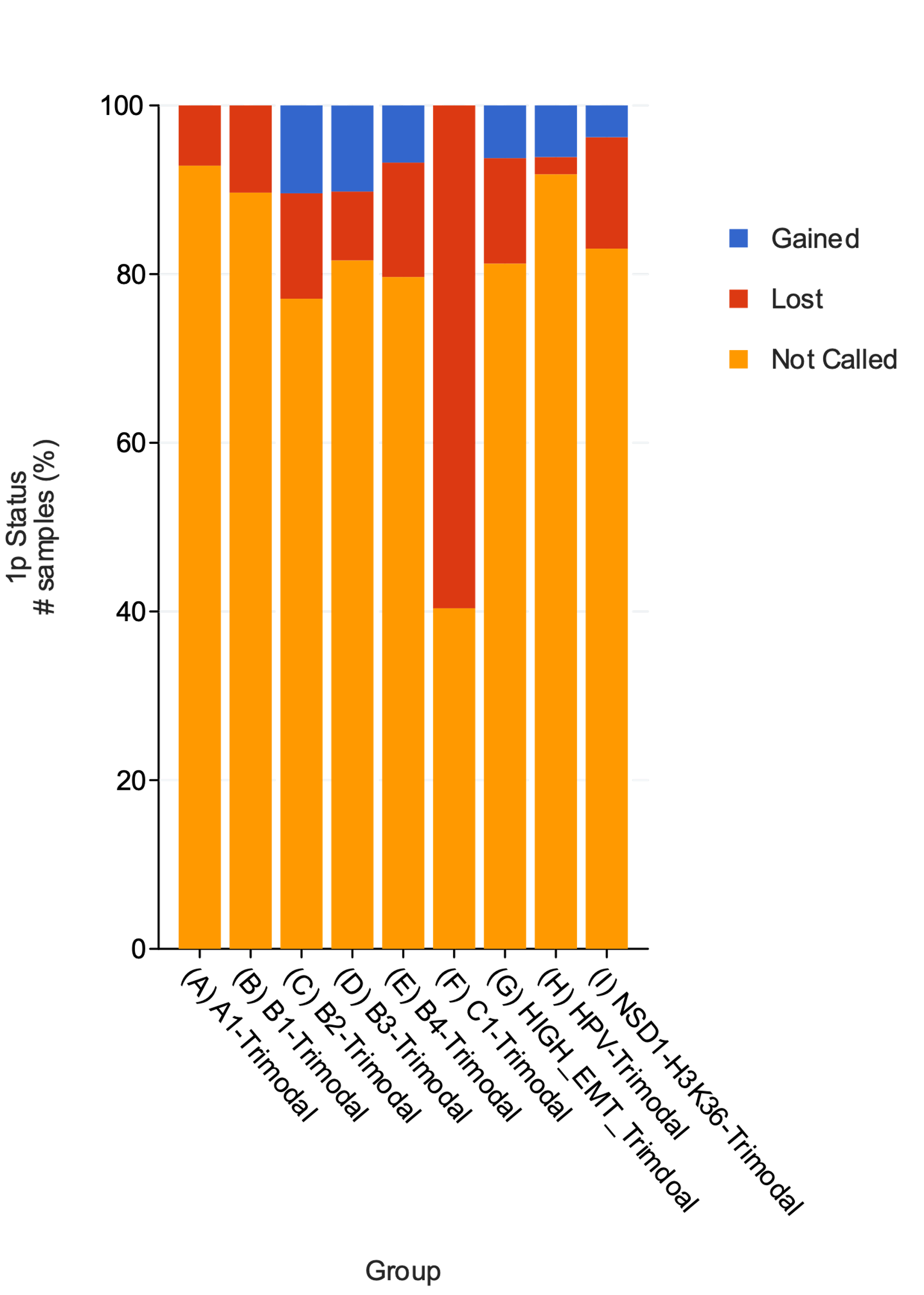


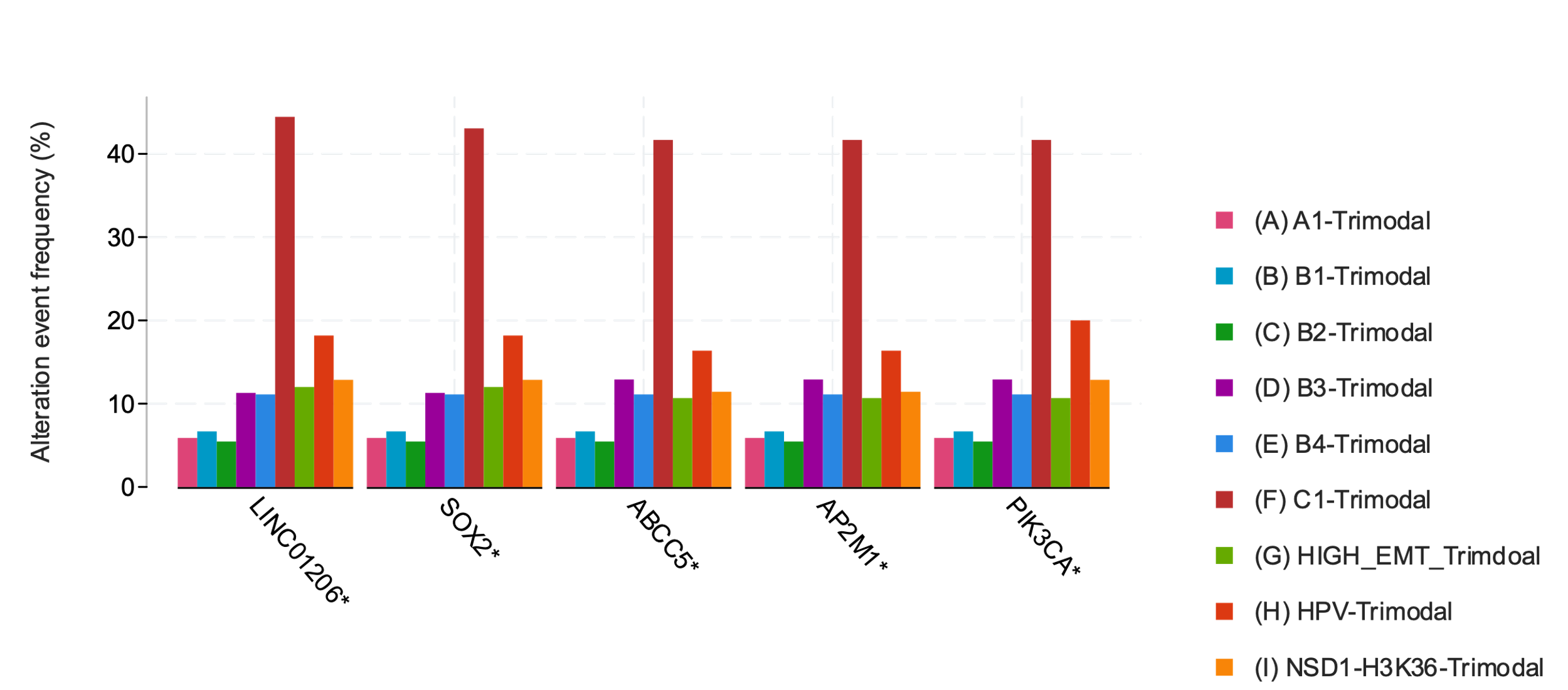

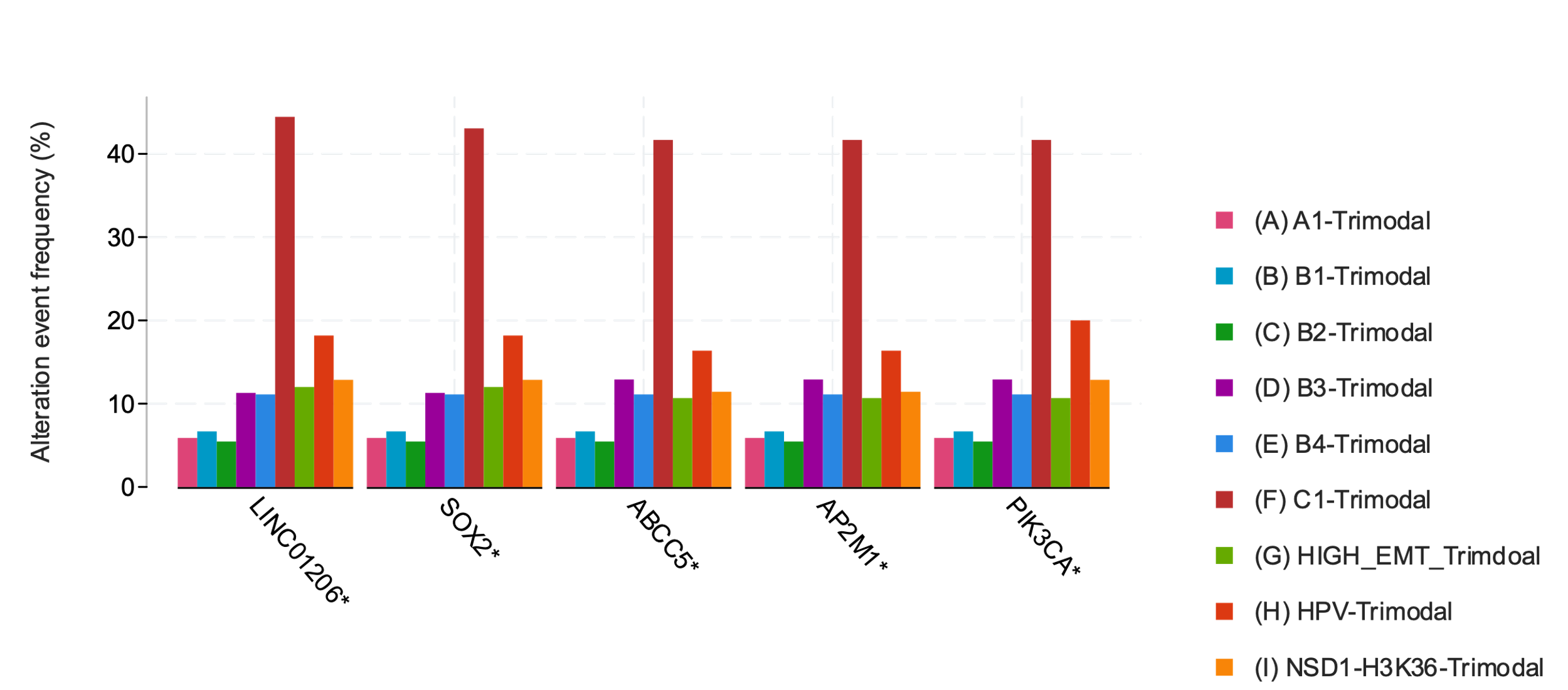


**Supplemental Fig 8. Genomic instability and mutations by spectral cluster.** Top: Fraction of the genome altered by sample. Middle: 1p and 4p alteration status. Bottom: Gene amplifications in chr3 q26.33-q27.1.


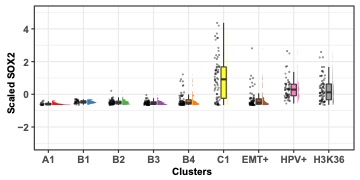


**Supplemental Fig 9. SOX2 Expression across spectral clusters.** Scaled TCGA-HNSC expression of SOX2 organized by spectral cluster calls.


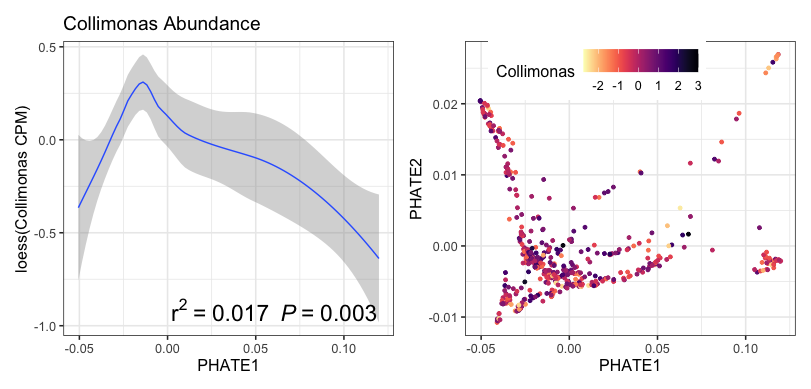

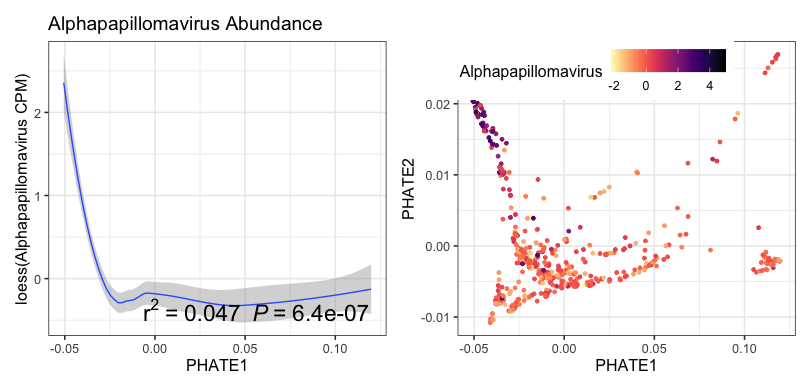

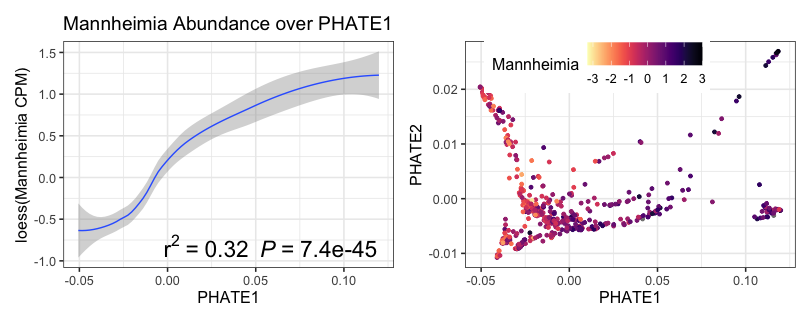

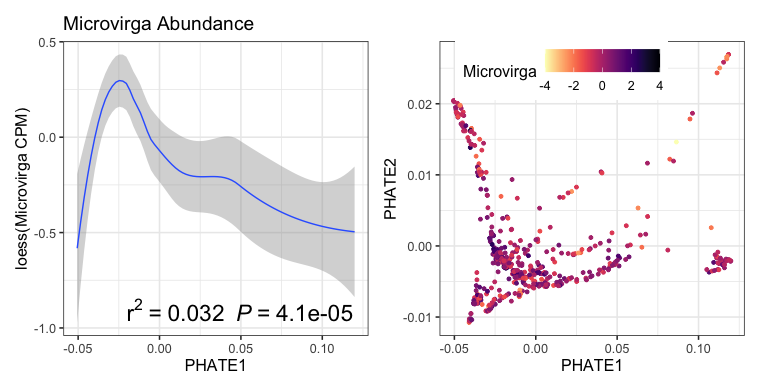

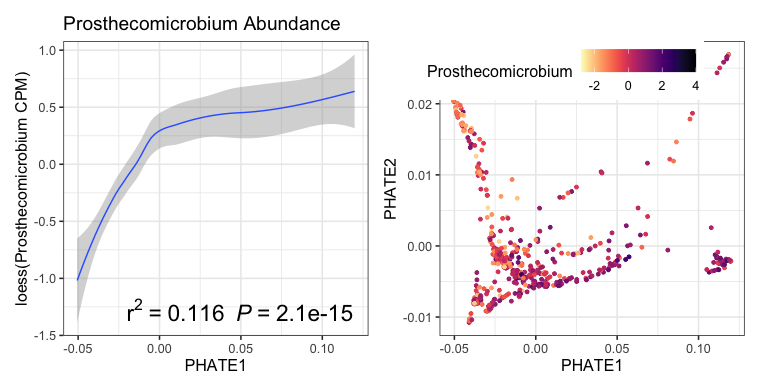

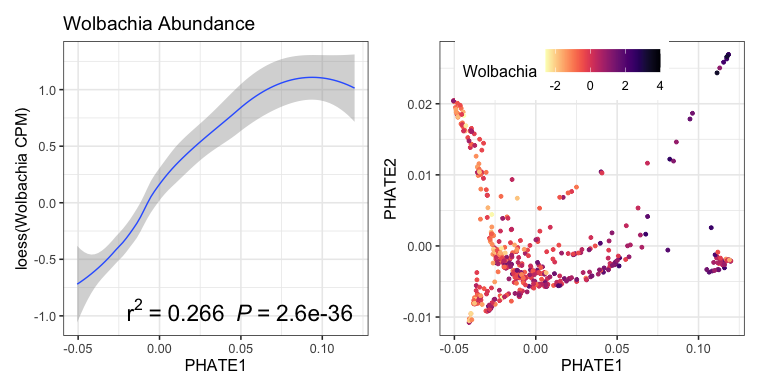


**Supplemental Fig 10. Microbiome across spectral clusters.** Scaled TCGA-HNSC expression of SOX2 organized by spectral cluster calls. Left: Loess curve smoothing of per-genus CPM estimate across the PHATE1 axis. Right: Scaled genus CPM in the PHATE projection.


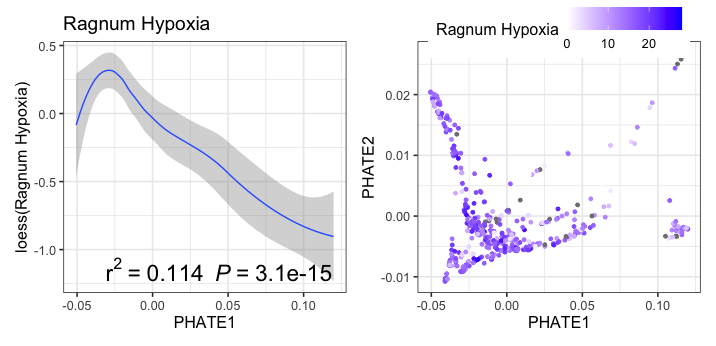

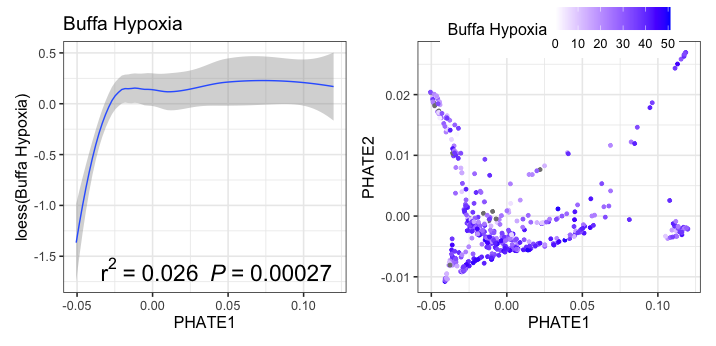

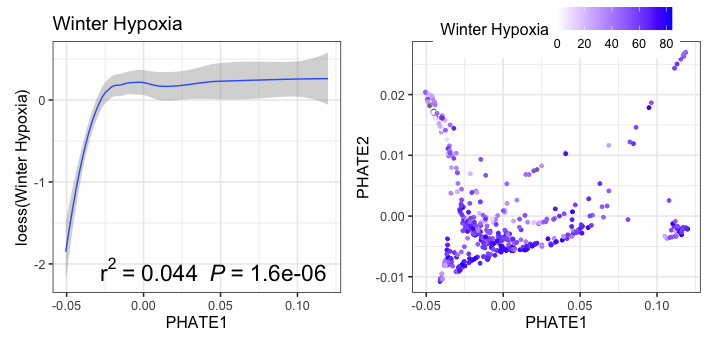
**Supplemental Fig 11. Estimations of Tumor Hypoxia state.** PHATE reduction of 514 HNSC samples with Winter, Buffa, and Ragnum hypoxia estimates across tumor samples. Left: Loess curve smoothing of hypoxic estimate across the PHATE1 axis. Right: Hypoxic score in the PHATE projection.


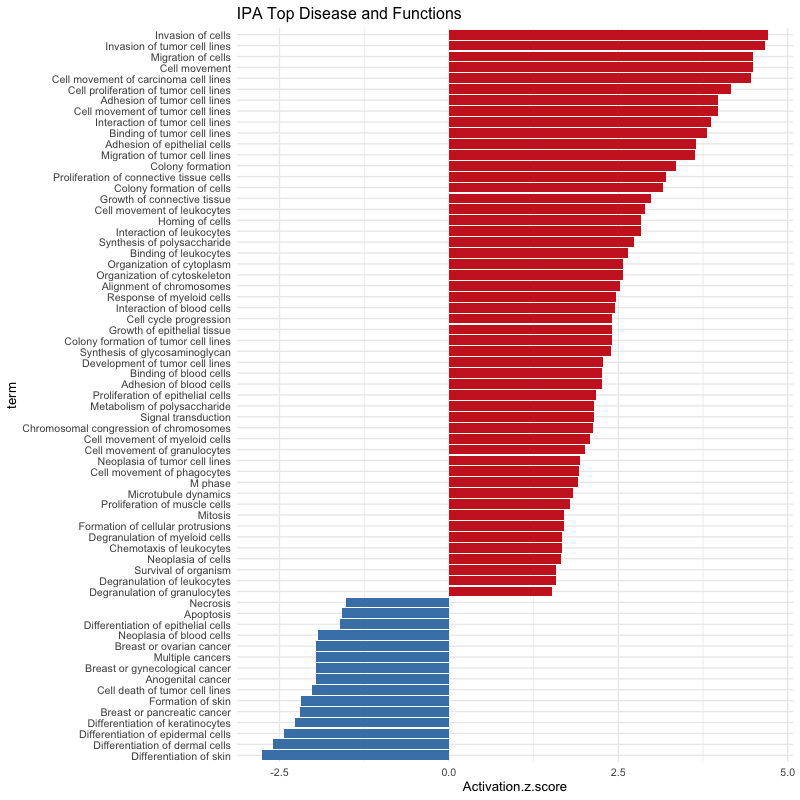


B4

B1

**Supplemental Fig 12. Ingenuity pathway analysis of differentially expressed genes between B1 and B4.** Activation Z. score of top differentially regulated pathways between B1 and B4.

**Supplemental Fig 13. TCGA-HNSC Neoplasm Histological Grade by Spectral cluster.** Per-cluster neoplasm histological grade assessed by the TCGA project. Data accessed via the CBioPortal.

**Supplemental Fig 14. TCGA-HNSC PHATE reduction and expression visualization of genes associated with partial-EMT.** Scaled gene expression plots for ITGA5, LAMC2, markers of partial-EMT, and KLK11 and S100A8, markers of epithelial differentiation.

**
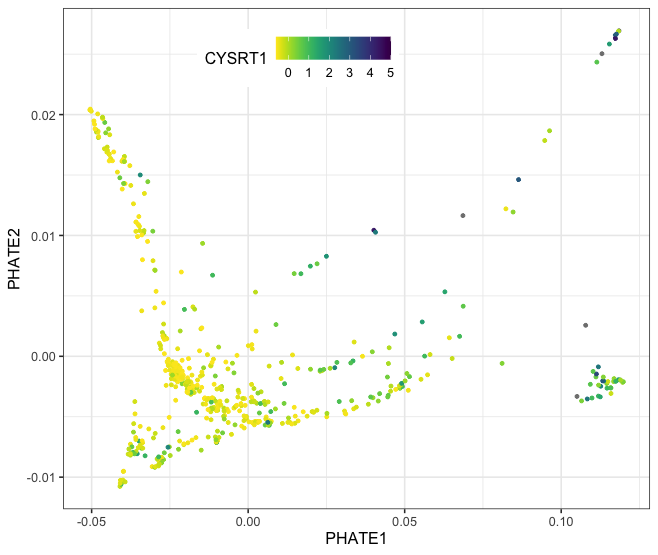
**

**Supplemental Fig 15. TCGA-HNSC PHATE reduction and expression visualization of CYSRT1, associated with superior prognosis.** Scaled gene expression plots for CYSRT1 (C9orf169).


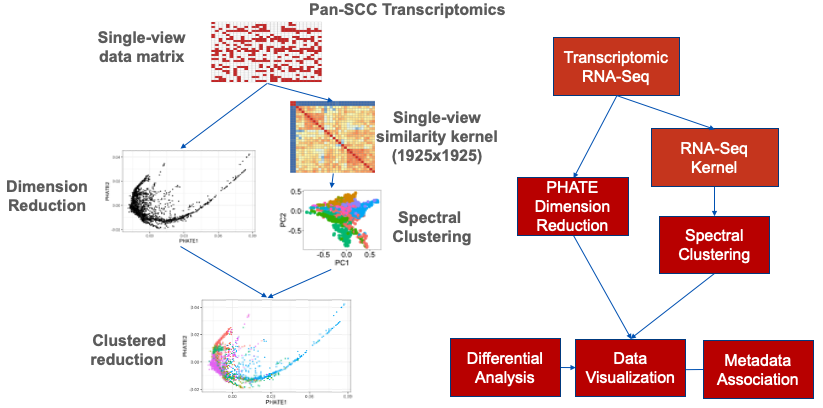


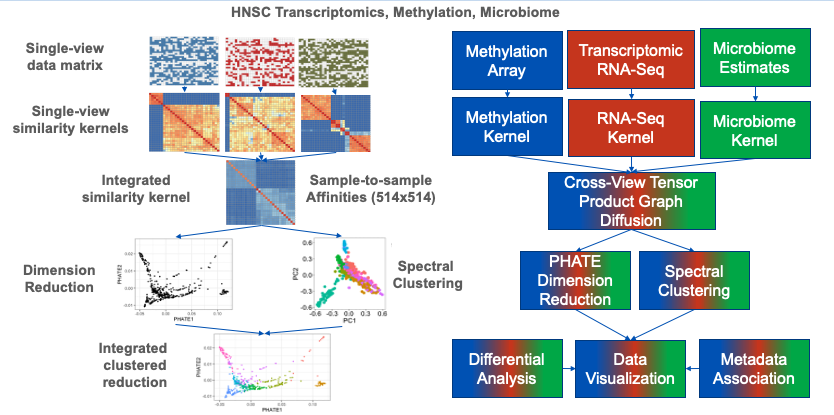


**Supplemental Fig 16. Analysis Schematic for single-view panSCC analysis and the trimodal TCGA-HNSC analysis.** Top: Analysis schematic for single-view reduction, and clustering of the 1925 TCGA panSCC cohort. Bottom: Analysis schematic for trimodal cross-view integration workflow for 514 TCGA-HNSC tumor samples.
